# A mixed-methods evaluation of a Polish international researcher mobility grant scheme

**DOI:** 10.1371/journal.pone.0327196

**Published:** 2025-06-25

**Authors:** Adam Ploszaj

**Affiliations:** Science Studies Lab and Centre for European Regional and Local Studies EUROREG, University of Warsaw, Warsaw, Poland; Ingenio CSIC-UPV, SPAIN

## Abstract

This paper examines the quantitative and qualitative effects of the first call of the Bekker Programme, funded by the Polish National Agency for Academic Exchange. The program supports the outgoing temporary international mobility of scholars. Its first call funded 152 scholars. The study employs a mixed-method approach, combining quantitative analysis using the interrupted time series method and qualitative analysis of final reports submitted by Bekker Programme beneficiaries. Quantitative data from the Web of Science database were analyzed to measure the number of papers published, the number of internationally co-authored works, and the number of citations for beneficiaries before and after the program’s implementation. The quantitative findings did not show a statistically significant increase in the number of publications or citations attributable to the program. However, there was a significant positive impact on international collaboration, as evidenced by an increase in internationally co-authored publications. Qualitative analysis of the beneficiary final reports revealed a broader spectrum of impacts, including enhanced scientific competencies, experience of different organizational cultures, networking and collaboration. The contributions of this article to the literature are threefold. First, empirical findings on the impact of a mobility program from a scientific periphery are presented, highlighting its possible transformative benefits. Second, a mixed-methods approach is employed that captures both objective quantitative effects and subjective outcomes, thereby acknowledging the multidimensional impact of mobility grants and offering practical insights for policymakers. Third, an empirical analysis of the eightfold mobility impacts framework is provided through a bottom-up coding analysis of qualitative data, demonstrating the framework’s applicability and robustness in a novel context.

## 1. Introduction

The international mobility of scholars is on the rise [1,2] and is attracting much interest as a research topic [3]. Funding schemes supporting international scholar mobility are staples in research policy toolboxes [4]. Programs supporting the mobility of scientists have been widely used in many countries and by many science funders, among which the most recognized are the Fulbright Program in the United States [5], the Marie Skłodowska-Curie Actions in the European Union [6], and the Humboldt Research Fellowship in Germany [7]. Studying the international mobility of scientists is also significant because it is an element of the broader and growing phenomenon of global mobility of highly qualified human resources [8] and the related negative effects (brain drain) and positive effects (brain gain) for the countries and regions participating in this circulation [9,10].

This article examines the quantitative and qualitative effects of the first call of the Bekker Programme supporting the temporary international outgoing mobility of scholars funded by the Polish National Agency for Academic Exchange (NAWA – the acronym comes from the Polish name of the agency: “Narodowa Agencja Wymiany Akademickiej”). NAWA was established in 2017 as a strategic initiative to address the low level of internationalization in Polish science and to strengthen the global engagement of Polish researchers. Bekker Programme was one of NAWA’s first major initiatives. It was named after Mieczysław G. Bekker, a graduate of Warsaw Technical University (1929) who fled Poland during World War II, worked at the University of Michigan and General Motors, and contributed significantly to the design and construction of the Lunar Roving Vehicle used in the Apollo 15–17 missions on the Moon. The programme was designed as an ambitious and well-funded mobility scheme, significantly expanding on a much smaller initiatives (“Mobilność” and “Mobilność Plus” programmes) previously run by the Polish Ministry of Science and Higher Education. The Bekker Programme had a significantly larger budget than its predecessors, which enabled the funding of a greater number of mobility grants. The program covered not only living expenses but also included a family allowance, making it more attractive to researchers with dependents. It was open to applicants from all fields of science and allowed mobility to any country. However, since the quality of the host institution was one of the evaluation criteria—alongside the quality of the applicant and the project—the program inherently favored collaborations with more prestigious and recognized institutions. Eligibility for the program was structured to facilitate broad participation. Applicants were required to be individuals employed by a Polish scientific or higher education institution, but they did not need formal institutional approval or a signature from their employer. This allowed researchers a degree of independence in pursuing international collaborations without bureaucratic constraints from their home institutions. By offering favorable financial conditions, broad eligibility, and a simplified application process, the Bekker Programme enhanced conditions for increasing the international mobility of Polish scholars.

The focus on the Bekker Programme is justified both by its significance for the internationalization of Polish science and by the unique data access made possible through collaboration with NAWA. As the agency was interested in evaluating the program’s effectiveness, it provided access to final reports submitted by beneficiaries upon completing their mobility. These reports include self-assessments of achieved outcomes, experiences, and future research plans, making it a unique (largely qualitative) source rarely available in studies on the impact of mobility programs.

The contribution of this article to the existing literature is trifold. First, the study uses mixed research approach, in which, in addition to measuring objective quantitative effects, subjective effects reported by the program beneficiaries are also taken into account. Recognizing the multidimensionality of the impact of mobility grants is important not only from a theoretical point of view but also in terms of the practical implications for institutions funding mobility grants. Second, an empirical test of the eightfold mobility impacts framework proposed by Netz et al. is provided by this study. Rather than adopting the categories a priori, a bottom-up coding approach was implemented to analyze the final reports submitted by the Bekker Programme beneficiaries. This method allowed the thematic categories to emerge organically from the data and subsequently be compared with the established framework. As a result, the applicability and robustness of the eightfold categorization are demonstrated within a novel empirical context. Third, this study adds to the literature by presenting empirical findings on the impact of a mobility program from a scientific periphery, an aspect rarely explored despite its significance. This is particularly relevant in the context of academic peripheries, where the benefits of mobility—such as knowledge transfer, networking, and access to research infrastructure—can be especially transformative.

## 2. Prior work and eightfold mobility impacts framework

The benefits of scientific mobility can be seen as stylized facts [11] in the science of science and in the practice of science policy. The dominant view is that scientific mobility benefits individual scientists, scientific institutions, and the general advancement of science. Many mechanisms are at work here: international mobility enables collaboration, the acquisition of new competencies and experiences, access to data and infrastructure, and the forging of novel ideas in new environments. In a systematic review of 96 empirical studies on the effects of mobility, Netz, Hampel, and Aman [12] distinguished eight impacts of mobility: (1) international networks, (2) scientific productivity, (3) occupational situation, (4) scientific impact, (5) competences and personality (e.g., language skills, intercultural and organizational competencies, self-confidence), (6) scientific knowledge, (7) access to research infrastructures and funds, and (8) symbolic capital (recognition, reputation). The authors found that most published studies reported positive impacts of mobility in the identified areas. Positive impacts were reported in 85% of studies concerning international networks, 56% of studies examining scientific productivity, 54% of studies focusing on occupational situations (however, the percentage was significantly lower after controlling for confounders), 65% of studies discussing scientific impact (measured by citation counts and journal ranks), 100% of studies concerning competencies and personality, 77% of studies examining scientific knowledge, 50% of studies focusing on access to research infrastructures and funds, and 75% of studies discussing symbolic capital. However, it should be noted that in the case of the last four categories, the number of publications analyzing a given issue was small: 13 for competencies and personality, 13 for scientific knowledge, 8 for access to research infrastructures and funds, and 8 for symbolic capital.

The papers published after the Netz et al.‘s 2020 review [12] have also mostly reported positive impacts of international mobility. Finocchi et al. [13], Kotsemir et al. [14], and Holding et al. [15] report positive effects on both scientific productivity and scientific impact. However, the positive effect identified by Holding et al. [15] seems to be largely driven by the prestige of inbound institutions. Tartari et al. [16] found a positive impact on productivity. Gu et al. [17] and Liu and Hu [18] reported positive effects on productivity and collaboration networks but no evidence of increased impact. While Zhao et al. [19] found no overall impact on productivity, they acknowledge various other impacts among disciplines. Finally, Bojica et al. [20] reported a positive impact on the career development of internationally mobile scholars.

Quantitative analyses of the impact of international mobility usually focus on: (1) mobility of a permanent nature or long-term relocation related to accepting a position at a new institution abroad, or (2) the effects of programs supporting short- or medium-term mobility lasting a few months to a couple of years (the Marie Skłodowska-Curie Actions global fellowships funded by the European Union can last up to 3 years). The second approach fits into the developing trend of using quasi-experimental methods to examine the effects of competitively awarded research grants. This body of literature uses methodological approaches that allow conclusions about causality to be drawn, revealing findings similar to those of the literature on the largely positive effects of international mobility. Many studies have reported both increased productivity and quality as a consequence of scholars receiving competitive research funding [21–34]. Others have found a positive impact on productivity but no or weak evidence for an effect on quality measured by the number of citations [35–39]. A limited number of studies found no impact on productivity or quality [40,41]. In addition, only a few published papers reported mixed, conditional, or ambiguous results [25,42,43].

To sum up, the recent literature on the impact of international researcher mobility focuses on relatively easy-to-measure effects (especially productivity measured by the number of publications) and generally shows a positive effect. It can be hypothesized that the positive increase in published works may, to some extent, result from publication bias (i.e., the tendency to publish positive and statistically significant results and not to publish insignificant results) [44]. Despite the rich literature on the subject, further exploration of this issue is still important. In particular, there is a need for approaches that go beyond simple measures of productivity and impact. Building on these considerations, the analytical framework of this study explicitly connects the quantitative and qualitative dimensions of scholar mobility under a unified conceptual lens. In particular, it draws on the perspective advanced by Netz, et al.—encompassing eight interlinked categories of academic mobility effects: (1) international networks, (2) scientific productivity, (3) occupational situation, (4) scientific impact, (5) competences and personality, (6) scientific knowledge, (7) access to research infrastructures and funds, and (8) symbolic capital. However, instead of merely verifying whether observed and self-reported outcomes align with predefined categories, this study will employ a bottom-up coding strategy expected to identify emergent themes that map onto—and potentially extend—key concepts in that eightfold framework. In so doing, it not only uses the Netz at al. framework but also assesses the framework’s utility in capturing the multifaceted outcomes of scholar mobility, thereby testing its appropriateness for guiding both empirical research and policy considerations in this domain.

## 3. Background information on the first call of the Bekker Programme

The first call of the Bekker NAWA Programme was announced in 2018. In the first Bekker Programme, NAWA received 519 applications, and funding was granted to 156 applicants. However, the final number of beneficiaries was smaller because four applicants did not sign the agreement. As a result, 152 projects were funded. The success rate in the first call of the Bekker Programme was 30.1%, a high ratio for a competitive research grant program.

The beneficiaries in the first call included 70 women (46.1%) and 82 men (53.9%). The youngest beneficiary was 28 years old, and the oldest was 64. The average age of the beneficiaries was 37.5 (standard deviation: 6.6), and the median was 36. The age distribution of the beneficiaries was right-skewed. Scholars aged 30–40 dominated, but there were also individual cases of older people aged 50–64. The beneficiaries included 101 people with doctoral degrees (66.4% of the beneficiary population), 45 people with habilitations (29.6%), and six professors (4%).

**Fig 1:**
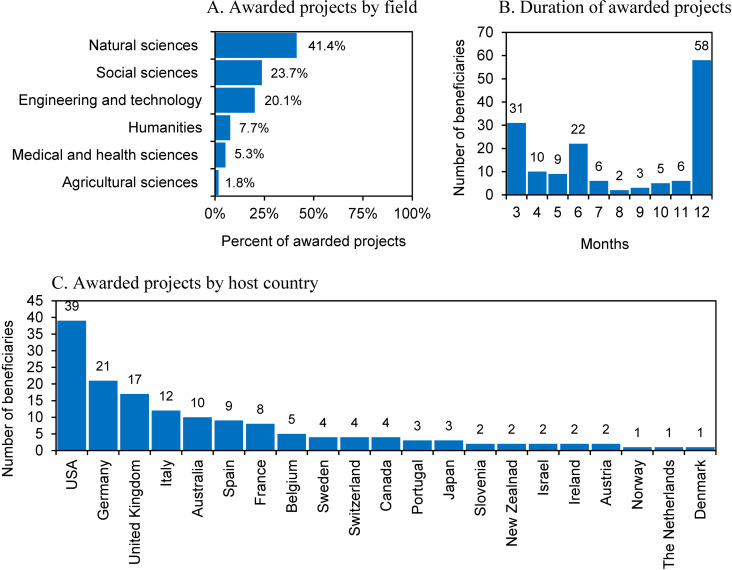
Selected data on the first call of the Bekker Programme: awarded projects by field (Panel A), by duration (Panel B), and by host country (Panel C).

The projects most often concerned natural sciences (41.4% of projects), social sciences (23.7%), and engineering and technical sciences (20.1%). Humanities (7.7%) and medical and health sciences (5.3%) were less frequently represented, and agricultural sciences (1.8%) was the least frequently represented field (Fig 1). The vast majority of the beneficiaries (130 individuals or 85.5% of all beneficiaries) were from higher education institutions. Another 19 individuals (12.5%) were employees of the Polish Academy of Sciences. Only three people (2%) represented research institutes. The beneficiaries came from 56 institutions. In terms of the number of beneficiaries, large public universities stood out. Jagiellonian University and the University of Warsaw (13 beneficiaries each) shared first place. Warsaw University of Technology (11) took second place, and the University of Lodz (10) came in third.

The beneficiaries of the first Bekker Programme planned visits to institutions located in 21 countries. There was a clear preference for European countries (60.5% of visits) and North America (28.3%). Mobility to Australia and New Zealand constituted 7.9% of trips. Asian countries were visited by 3.3% of the beneficiaries, although it should be noted that only two such countries were visited: Japan (three visits) and Israel (two visits). In the first call of the program, there were no visits to Africa or South America.

By far, the most frequently chosen country was the United States of America (USA); 39 beneficiaries planned to implement projects there. The second most frequently chosen destination was Germany (21 beneficiaries), and The third most popular was the United Kingdom (17). It is worth noting that among the European countries visited, countries located to the west of Poland dominate (Fig 1). The destinations of the beneficiaries suggest that successful applicants chose countries in the “scientific center” (i.e., countries with well-developed science systems and institutions) as places to implement their projects [4].

The vast majority of the institutions visited were universities. Non-university research institutions accounted for only 15.5% of host institutions. Among the institutions visited were the most prestigious universities globally. The beneficiaries of the Bekker Programme visited nine of the institutions listed among the top ten in the Academic Ranking of World Universities (ARWU) in 2019. These were the University of California, Berkeley, the California Institute of Technology (Caltech), the University of Columbia, Harvard, Massachusetts Institute of Technology (MIT), Oxford University, Princeton, and Stanford. The University of Chicago was the only university from the top 10 in the ARWU that was not among the destinations of the beneficiaries of the first call of the program.

In total, 15 beneficiaries implemented their projects in institutions listed among the top 10 in the ARWU. This represented 11.5% of beneficiaries who implemented projects in higher education institutions (only higher education institutions are classified in the ARWU, so beneficiaries who visited institutions of other types are not included in this calculation). Every fourth beneficiary (25.4%) implemented their project in a higher education institution listed among the top 50 in the ARWU. This indicator reached 44.6% in relation to the top 100 places in the ARWU.

In the first call of the Bekker Programme, applicants could apply for funding for mobility lasting from 3 to 12 months. Among the projects granted, most involved one-year visits (58 beneficiaries (38.2%)). The second most popular category of mobility length was three-month stays. Such projects were implemented by 31 beneficiaries (20.4%). The third category was six-month stays (22 beneficiaries (14.5%)). Trips lasting 4–5 or 7–11 months were carried out significantly less often than trips lasting 3, 6, or 12 months (Fig 1). Generally, two dominant groups can be distinguished: the one-year trips discussed earlier (38.2%) and trips lasting three to six months, which constituted almost half of all projects (47.4%).

## 4. Data and methods

This study used a mixed research framework. First, data on the publications of program beneficiaries indexed in the Web of Science database were analyzed using the quasi-experimental interrupted time series method. This allowed for a more objective (although limited to easily measurable aspects) analysis of the program’s impact. Second, using a qualitative approach, final reports submitted by beneficiaries of the first call of the Bekker Programme were analyzed, providing qualitative insights into the program’s impact. This study has been reviewed and approved by the EUROREG Ethical Review Board (approval reference number: KEB-EUROREG-20–1).

The Web of Science database was searched for published works of all beneficiaries of the first call of the Bekker Programme. Data on all beneficiaries of the first call of the Bekker program are publicly available on the NAWA website (www.nawa.gov.pl) and include: name and surname, home institution, host institution, host country, duration of the scholarship, amount of the scholarship, and title of the project. Web of Science search was carried out in May 2024 to ensure that the collection would include full data up to the end of 2023. The search was conducted manually to ensure high data quality, which is particularly important with a relatively small sample (but, on the other hand, it was the full population of beneficiaries of the first call of the program). Citation data were re-downloaded in early June 2025 to accommodate longer citation windows, specifically the five-year citation window recommended for social science and humanities data [45–47]. The following indicators were calculated based on the raw data: number of publications, number of internationally co-authored publications (as a proxy for international collaboration), and number of publications acknowledging Bekker Programme funding. Publication counts were calculated using both whole counting and fractional counting methods. Citation counts (average citations per publication) were calculated in four citation window variants: two-year, three-year, four-year, and five-year citation windows. All variables were calculated per year, starting from 2014 and ending in 2023. This timeframe covered five years (2014–2018) before the announcement of the first Bekker Programme and five subsequent years (2019–2023). The year 2019 was treated as the first call in which program effects could occur.

**Fig 2:**
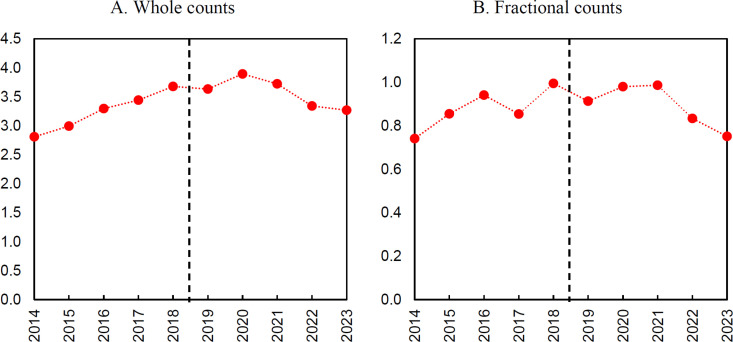
Number of publications.

**Fig 3:**
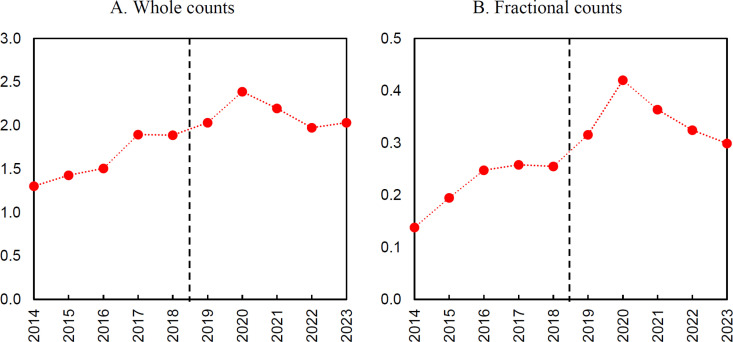
Number of internationally co-authored publications.

Quantitative data were analyzed using the interrupted time series approach [48]. This quasi-experimental method allows for impacts to be identified in the absence of control group data (for legal reasons, access to information about unsuccessful applicants for the analyzed program was not possible). Interrupted time series assumes that the intervention causes a change in a long-term trend. In our case, the change caused by the Bekker Programme grants may be reflected in a disturbance in the pattern of analyzed indicators in the period after the grant was received (i.e., after 2018). A fundamental assumption of the interrupted time series (ITS) design is the presence of a consistent pre-intervention trend. In the present analysis, this assumption is met, as evidenced by the clear linear growth trajectories observed in the pre-intervention period (see Figs 1–3). Of course, the lack of a control group makes the interrupted time series a relatively weak quasi-experimental method compared to alternatives with control groups. However, its conclusions can be instructive, although great caution should be exercised in their interpretation [49]. Furthermore, It is important to note that the absence of a control group is particularly problematic when the analysis indicates the presence of an effect, as it limits the ability to rule out alternative explanations. In the presented case, the ITS results indicate overall no significant effect, which reduces the problem of the lack of a control group – this is because any observed change in the outcome would necessitate that the control group exhibited not a stable trend but a deviation, which is an unlikely scenario.

The analysis used interrupted times series with panel data (because there are many individual beneficiaries) and fixed effects, which allowed for heterogeneity between individual beneficiaries (derived from, among other things, different disciplines or different career stages) to be taken into account. Modeling was performed using the XTITSA [50] package for STATA. Eight model specifications were estimated to assess the impact of the Bekker Programme, each corresponding to a different outcome variable. The analysis included several dependent (outcome) variables to capture this impact. The number of papers published was measured in two versions: whole counting and fractional counting. Similarly, the number of internationally co-authored publications was also measured using whole and fractional counting methods. Additionally, the average number of citations per paper was considered across four citation windows: two years, three years, four years, and five years. The key independent variable used in the analysis was a binary variable—named “Treatment”—indicating the period before and after the first call of the Bekker Programme: 0 for years up to 2018 and 1 for 2019 and onwards. The “Publication year” variable denoted the year in which the publications were released, ranging from 2014 to 2023. Gender was represented by a binary variable named “Female,” coded as 1 for female and 0 for male. The “Age” variable, representing the age of the beneficiary at the time of the grant award, was included to account for potential career-stage effects. The field of science variable distinguished between social sciences and humanities (coded as 1) and other fields (coded as 0), and was named “Social Sciences and Humanities” with the short name “Soc&Hum” in the regression tables. To account for the possible influence of institutional quality and prestige, a dummy variable was introduced to indicate whether the host institution was ranked among the top 10 in the Academic Ranking of World Universities. The variable was named “ARWU10” in the regression tables. To account for the possible influence of institutional quality and prestige, the variable indicating whether the host institution was ranked among the top 10 in the Academic Ranking of World Universities (coded as 1) and those hosted elsewhere (coded as 0), and was named “ARWU10” in the regression tables. The “Host country” variable was also included in all specifications as a dummy but was not reported in the regression results table. All models employed robust standard errors clustered by individuals to address the potential lack of independence and autocorrelation in the observations for particular individuals. The regression equation used in the analysis is as follows:


$$\begin{array}{cc} Y_{ti} & =\;\beta_{0}+\;\beta_{1}{Time}_{ti}\;+\;\beta_{2}{Treatment}_{ti}\;+\;\beta_{3}{{Time}_{ti}Treatment}_{ti}\;+\;\beta_{4}{Female}_{i}\;+\;\beta_{5}{Age}_{i}\;+\;\beta_{6}{Soc\&Hum}_{i}\;\\ & +\;\beta_{7}{ARWU10}_{i}\;+\;\sum \beta_{8c}{Country}_{ic}\;+\;\varepsilon_{i} \end{array}$$

where Y represents the dependent variables (as defined above), measured annually for each beneficiary, Time is a continuous variable capturing the year, Treatment is a binary indicator for the post-intervention period (1 for 2019 and onwards, 0 otherwise), and Time*Treatment captures the change in trend following the intervention. The remaining independent variables represent time invariant variables as defined in the previous paragraph. The error term ε is clustered at the individual level to account for within-subject correlation over time.

In the second part of the study, final reports submitted to NAWA by beneficiaries were analyzed. The reports contain personal data allowing the identification of beneficiaries—for this reason, making this data public is not possible. The data was accessed in the period December 28, 2020 – April 13, 2021. The qualitative analysis of this very descriptive and largely unstructured data was conducted using the MAXQDA software. The texts of the reports were coded bottom-up, without pre-defined categories, and then aggregated into analytical categories. This approach broadly corresponds to the assumptions of grounded theory [51]. Its application was aimed at detecting effects other than the obvious ones (such as the number of publications) and identifying justifications and narrative examples provided by beneficiaries. This method also requires caution in interpretation, given the challenges associated with self-reporting and the related fact that beneficiaries may have exaggerated the effects in attempting to meet the (imagined) expectations of the funding institution.

## 5. Results

### 5.1. Observed bibliometric effects

From 2014 to 2023, the beneficiaries of the first call of the Bekker Programme published an average of 3.4 publications per year (it should be mentioned that only publications included in the Web of Science were considered in this section of the analysis). When the number of co-authors by fractional counting is taken into account, the average is 0.9 publications per year. In the case of publications with foreign co-authors, the average number of publications is 1.9 per year in the whole counting version and 0.3 in the fractional counting version. The average number of citations of articles is 19.3 in the two-year, 34 in the three-year, 46.8 in the four-year, and 57.2 in the five-year citation windows. The variables are clearly right-skewed, as evidenced by the median value being significantly lower than the average (Table 1). The maximum value for the number of publications and citations can be attributed to the presence of two beneficiaries in the sample who were researchers working in the field of particle physics, where large research teams publishing dozens of articles per year are the norm.

**Table 1:** Descriptive statistics.

		Average	SD	Median	Min	Max
Publications per year (2014–2023)	Whole counts	3.4	6.1	2	0	85
	Fractional counts	0.9	1.0	0.6	0	11
Internationally co-authored publications per year (2014–2023)	Whole counts	1.9	5.7	1.0	0	85
	Fractional counts	0.3	0.5	0.1	0	4
Citations per publication per year (2014–2023) in citation windows	Two-year window	19.3	67.7	4.0	0	1133
	Three-year window	34.0	116.3	8.0	0	1982
	Four-year window	46.8	159.4	11.0	0	2723
	Five-year window	57.2	200.0	14.0	0	3516
Publications acknowledging Bekker funding, cumulative in years 2019–2023	Whole counts	2.3	3.4	1.0	0	21
	Fractional counts	0.7	1.0	0.3	0	6

The annual data for the analyzed indicators (Figs 2 and 3) shows no interruption around the cut-off point between 2018 and 2019, or in later years, when the impact of funding through the Bekker Programme could have occurred. Instead, a continuation of earlier trends is present. Regarding the number of publications, it is noticeable that after a period of growth, there is a decrease in the average number of publications in the last two years covered by the analysis. This also applies to the number of publications with international co-authors. However, it should be noted that the variable for the average number of articles with foreign co-authors in the fractional counting variant stands out from all other analyzed variables in that a clear jump in value is visible in the period 2018–2020. This suggests that the increase is related to the impact of international scholar mobility.

**Fig 4:**
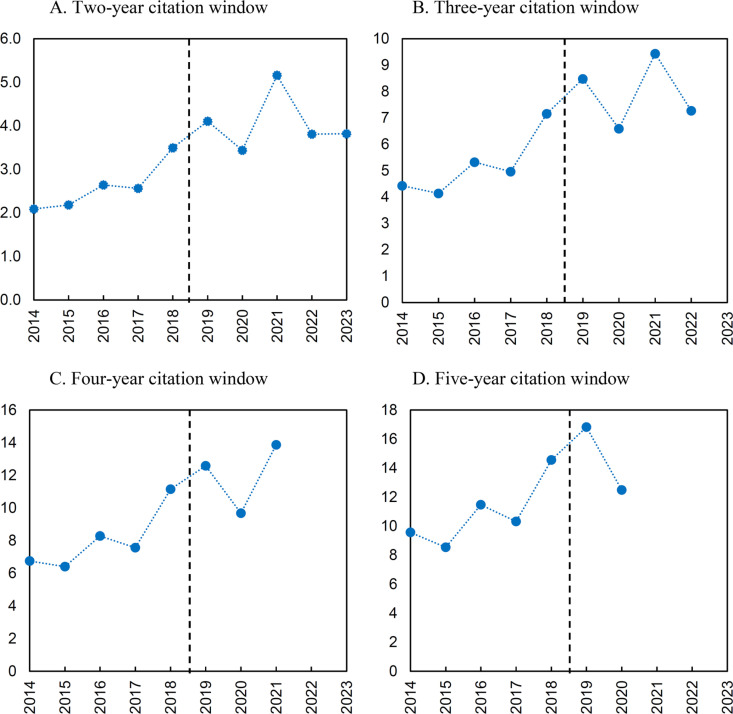
Average number of citations per publication in various citation windows. * To ensure comparability of data across different citation windows, annual data are presented only for years that allow the entire citation window to be included. For example, for a five-year citation window, the last year that allows the entire five-year period to be included is 2020 (a citation window covering the period 2020–2024).

Annual data on the average number of citations of publications published by the beneficiaries of the Bekker Programme reveal a clear upward trend (Fig 4), albeit with large fluctuations, especially in recent years, which means that the random factor is important here and one should be cautious when drawing conclusions based on these data. The data do not show any clear disruption after the Bekker grants were awarded.

**Fig 5:**
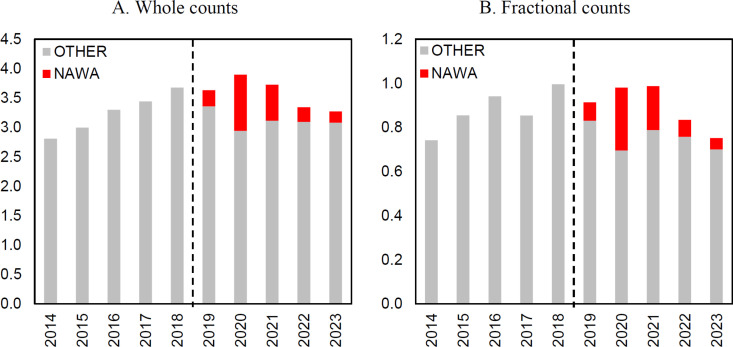
Total number of publications and publications acknowledging Bekker Programme support.

The number of publications that acknowledged funding from the Bekker Programme is significant. A total of 347 such publications published by the beneficiaries of the first call of the Bekker Programme in 2019–2023 were identified in the Web of Science database. This gives an average of 2.3 publications per beneficiary wholly counted or 0.7 fractionally counted (cumulative in the period 2019–2023). Papers indicating funding from NAWA began appearing in publications in 2019 (on average 0.28 papers per beneficiary). The largest number of papers mentioning this funding appeared in 2020 (0.95). In the following years, the number of papers acknowledging support from the Bekker Programme decreased (0.61 in 2021, 0.25 in 2022, and 0.19 in 2023). A similar pattern is visible in the fractional counting: 0.08 in 2019, 0.29 in 2020, 0.20 in 2021, 0.08 in 2022, and 0.05 in 2023. Papers acknowledging funding from Bekker Programme grants constituted only a minor fraction of the beneficiaries’ annual publications between 2019 and 2023, accounting for 8%, 24%, 16%, 7%, and 6% of all studies in the whole counting variant, and 9%, 29%, 20%, 9%, and 7%, in the fractional counting variant. The chart in Fig 5 shows annual publications divided into those financed by the Bekker Programme and other publications, suggesting a substitution effect. In other words, published articles resulting from research projects financed by the Bekker Programme may “replace” or “take the place of” publications that might have been created anyway if the trend in the productivity level of the beneficiaries from before they participated in the program had continued.

The interrupted time series regression analysis largely confirms the observations based on the annual data. The regression results do not provide evidence of a statistically significant impact of receiving the Bekker grant on productivity measured by published papers counted wholly and fractionally, nor on scholarly impact measured by the average number of citations in different citation windows (Table 2). The only statistically significant effect identified—exclusively in the fractional counting approach—relates to the number of papers with foreign co-authors (The robustness of this result is supported by the analysis based on data for a shorter period, i.e., 2015–2022, see Table 1A in the S1 File attached to this file). In the whole counting approach, the coefficient is also positive, but the effect is not statistically significant. This result is not surprising; ultimately, the beneficiaries had real opportunities, and perhaps even an obligation, to establish and continue collaboration with foreign scholars, especially those from the institutions to which they temporarily relocated.

**Table 2:** Regression results.

Outcome variable	(1)	(2)	(3)	(4)	(5)	(6)	(7)	(8)
	Papers published		Internationally co-authored papers		Average citations per paper in citation windows			
	Whole	Fractional	Whole	Fractional	2-year	3-year	4-year	5-year
Treatment	−0.071	−0.042	0.112	0.063^**^	0.554	0.971	0.378	2.415
	(0.240)	(0.080)	(0.181)	(0.031)	(0.457)	(0.955)	(1.752)	(2.804)
Pub. year	0.218^***^	0.051^**^	0.164^***^	0.030^***^	0.319^***^	0.629^***^	0.995^***^	1.173^***^
	(0.051)	(0.020)	(0.040)	(0.008)	(0.105)	(0.196)	(0.309)	(0.406)
Soc&Hum	−2.509^***^	−0.221^**^	−1.638^***^	−0.154^***^	−1.521^***^	−2.433^**^	−3.622^**^	−4.117^*^
	(0.609)	(0.110)	(0.571)	(0.043)	(0.483)	(0.972)	(1.599)	(2.261)
ARWU10	0.582	0.020	0.435	0.014	0.864	1.505	1.762	1.694
	(0.575)	(0.146)	(0.542)	(0.067)	(0.568)	(1.147)	(1.902)	(2.762)
Female	−1.429^*^	−0.296^***^	−1.142	−0.154^***^	−0.516	−1.106	−1.861	−2.789
	(0.748)	(0.105)	(0.739)	(0.044)	(0.495)	(0.997)	(1.612)	(2.413)
Age	−0.053	0.022^***^	−0.066	0.004	0.050	0.099	0.170	0.231
	(0.070)	(0.007)	(0.069)	(0.003)	(0.035)	(0.070)	(0.122)	(0.184)
Host country (dummy)	✓	✓	✓	✓	✓	✓	✓	✓
Observations	1,520	1,520	1,520	1,520	1,520	1,368	1,216	1,064

Robust standard errors in parentheses

*** p < 0.01,

** p < 0.05,

* p < 0.1

Across all model specifications, scholars in the social sciences and humanities (Soc&Hum) consistently exhibited lower values for the dependent variables compared to their counterparts in other fields, with statistically significant negative coefficients across all outcomes. The variable indicating affiliation with an institution ranked among the top 10 in the Academic Ranking of World Universities (ARWU10) showed no discernible effect on any of the examined indicators, suggesting that the prestige of the host institution did not systematically translate into increased research output or impact. Age appeared to be largely irrelevant to research outcomes, with no consistent effects across models. Finally, female beneficiaries exhibited a generally negative effect, particularly in terms of publication productivity, as evidenced by significant negative coefficients in both whole and fractional counting approaches. However, no statistically significant differences were observed in citation-based impact measures, indicating that while female scholars may publish less, their work is cited at comparable rates.

Further analyses of sub-samples reveal that the above-discussed effect on fractionally measured internationally co-authored papers is significant among younger beneficiaries but not among older ones, significant among beneficiaries affiliated with the exact, technical, and medical sciences but not among those in the social sciences and humanities, and significant among beneficiaries who were more productive prior to the grant but not among those less productive. Disaggregation based on gender indicates no difference. These findings suggest that the program’s influence on fostering international collaboration—measured by co-authored publications—operates primarily among younger, already more productive researchers in the exact, technical, or health sciences. Regarding other outcome variables, analyses conducted across subgroups defined by gender, age, field of research, and pre-grant productivity suggest no evidence of heterogeneity, as no significant impacts were observed overall (see Tables 2A–9A in the S1 File for further details).

### 5.2. Self-reported effects

This section discusses the effects of participation in mobility sponsored by the Bekker Programme, as described in the final reports submitted by the beneficiaries of the first call. Based on a qualitative analysis of the reports, nine groups of effects were distinguished, which are discussed below.

#### 5.2.1. Publications.

The most apparent and expected outcome of research projects is publication of research papers. Almost all the beneficiaries of the first Bekker Programme declared that they had published or at least prepared one paper as a result of their stay in a foreign institution. This applied to 98.6% of projects (138 projects with declared publications out of 140 projects with final reports). Only two beneficiaries did not publish. Both cases involved stays at prestigious universities in the USA and were in the field of natural sciences (biology). One of the scholars explained:

Due to […] unpromising results of preliminary research resulting in a change in the direction of further research, it was not possible to present the project results in the form of a scientific publication at this time.

The most common form of publication was articles in journals, and conference proceedings. Almost all the beneficiaries stated that they had prepared, or were preparing, scientific articles as a result of their projects (137 people, which is 97.9% of the submitted reports). Among the beneficiaries who revealed mobility effects in the form of articles, about a third mentioned one article. The remaining two-thirds declared that they had worked on two or more articles, although occasionally, there were declarations of five or more articles (less than 10% of the program participants). Sixteen beneficiaries (11.4%) stated that the result of their participation in the program was the preparation of a monograph (mostly planned for publication in foreign publishing houses). The beneficiaries usually indicated that they had prepared a portion (chapter, section) of the monograph or only its assumptions (outline, publishing proposal), and work on the manuscript would continue. This is understandable because a large amount of work is usually needed to create a monograph, and the entire process generally takes years rather than months.

Two people declared they had prepared academic textbooks, and another three mentioned edited volumes. For two respondents, the effect of scholastic mobility involved participation in planned special issues of scientific journals in cooperation with people from the host institutions. Only eight people (5.7%) reported publication in the form of chapters in edited volumes as a beneficial effect of the Bekker Programme. The surprisingly small number may be related to the low ranking of this type of publication in the evaluation of scientific disciplines in Poland [52,53] and to the relatively low importance of chapters when applying for grants at the Polish National Science Centre (the main public funder of basic research in Poland). This situation may also be partially explained by the relatively small representation of social sciences and humanities among the beneficiaries of the first Bekker Programme; book chapters are more popular research outcomes in these fields than in the natural, technical, and medical sciences. In the analyzed set, almost all (seven out of eight) declarations regarding chapters came from beneficiaries in the social sciences or humanities.

#### 5.2.2. Conferences.

About two-thirds of the beneficiaries declared beneficial effects in the form of conference, seminar, or workshop presentations. Active participation in the form of an oral presentation or a conference poster presentation was most often declared. Four beneficiaries received invitations to deliver plenary or keynote speeches. Ten percent of beneficiaries were involved in arranging scientific meetings (seminars, conferences, workshops) in cooperation with the host center or people they met during the program. The conference activity of the beneficiaries was clearly diversified by discipline. All the beneficiaries who implemented projects in the field of humanities and agricultural sciences indicated participation in conferences or other scientific meetings as a result of the Bekker Programme. The majority of the beneficiaries in the medical and health sciences (almost 89%) and social sciences (73%) participated in such conferences. The beneficiaries representing natural sciences (55.2%) or engineering and technical sciences (54.8%) had the lowest conference involvement.

#### 5.2.3. Grants.

Almost three-quarters of the beneficiaries (72.1%) declared that they had prepared new grant applications as a result of participating in the program. Some beneficiaries made general statements about the preparation of new projects; others provided the names of grant schemes and funding agencies to which they had applied, or would apply, for funding. Several respondents (less than 10%) stated that a new grant had been obtained. Four beneficiaries had applied for a grant but did not receive funding. The beneficiaries mostly wrote about one project, but occasionally, they had applied for more grants. In addition, several beneficiaries stated that during the program, they were invited by the host unit to contribute to an implemented or planned grant. Beneficial effects of mobility in the form of new grant applications were most often declared by beneficiaries in engineering and technical sciences (90.3%). Medical and health sciences also stood out (77.8%). Approximately two-thirds of beneficiaries in the remaining fields reported the preparation of new grants.

#### 5.2.4. Patents and opportunities for commercialization.

Six beneficiaries declared they had been involved in patent applications. Some of these declarations pertained to applications in the process of development or applications planned for the future. However, it should be emphasized that two beneficiaries clearly stated that they had filed patent applications. One of these declarations described the filing of three applications, and the other discussed six. In the latter case, the patents were filed jointly by Polish and foreign institutions, which indicates that they were a direct result of cooperation. Some beneficiaries stated that the patent filing procedure contributed to delays in publishing study results in the form of scientific papers (disclosure of the subject of the patent application would have made it impossible to obtain patent protection).

Three beneficiaries highlighted commercialization potential and opportunities for establishing cooperation with businesses related to their project results. One beneficiary mentioned the names of two companies (a company from the USA and a large listed company from Poland) interested in cooperation. Comparing these declarations with the declarations concerning patents, we can conclude that nine projects may have effects in the area of commercialization of research results.

#### 5.2.5. Collaboration and networking.

All reports highlighted the beneficial effects of collaboration with host institutions. However, this observation is inconsequential because a certain level of collaboration was necessary to implement all the projects and obtain confirmation of visits to the host institutions. The beneficiaries were highly optimistic about the possibility of continuing cooperation, with 95.8% claiming that they would very likely continue cooperation with their host institutions. Only six people (4.2%) claimed that further cooperation was unlikely or moderately likely. The stays of these six people who were less certain about continuing cooperation were of different lengths: 3, 6, and 10 months (one person each) and 12 months (three people). This distribution suggests that a stay that is too short cannot be the main reason for a less positive attitude to continuing cooperation. Among the explanations for indicating a moderate probability of continuing cooperation, the beneficiaries mostly mentioned two factors: the predominance of independent work during the stay and the high workload of researchers in the host institutions, which made the host researchers relatively inaccessible to the visitors.

Several detailed and sometimes less apparent aspects of cooperation emerged from the statements of the beneficiaries. Some beneficiaries emphasized that some of the collaboration in their host institutions was planned, and some arose through chance meetings with people from other organizational units of the host institutions or visiting researchers. Two of the respondents described this aspect as follows:

At the same time, as part of the internship, I had the opportunity to participate in a number of seminars and informal scientific meetings […]. One such scientist was Prof. [name, surname]. The effect of my participation in the above-mentioned seminar is the established scientific cooperation with Prof. [name, surname] and the planned application for an internship at [large university in Japan], workplace of the professor I met.

And another beneficiary stated:

During the internship, I established new professional contacts, not only with researchers from [host unit] but also from other research centers in Great Britain (such as Oxford University, City University of London) and other parts of the world (e.g., Capital Medical University [China], Pusan National University [South Korea], or Tohoku University [Japan]), representing various fields of science.

When describing the beneficial effects of their projects, about half of the beneficiaries emphasized the importance of networking and expressed the hope that it would translate into future collaborations. The development of networks of contacts primarily concerned relations with scientists, but there were also some indications of establishing contacts with people from the business sector or public administration.

Four beneficiaries received offers to extend their stays at their host institutions by several months to complete their projects. Host institutions financed these extensions, which can be interpreted as a signal of appreciation for the collaborations with the beneficiaries. The cases of extensions of stays at the expense of host institutions exclusively concerned beneficiaries working in the field of experimental sciences (one project each in the fields of biochemistry and medical biochemistry, and two in the field of physics).

About 10% of beneficiaries declared the continuation of collaborations with their host institutions in the form of visits in the forthcoming years. The level of specificity of these declarations varied from indicating that the beneficiary had an invitation from the foreign institution for a specific date and on specific terms to loose plans for trips dependent on a new grant being obtained.

Another dimension of extending collaborations with host institutions is the formal maintenance of a foreign affiliation (referred to as a faculty fellow, affiliate, collaborator, etc.). Such offers were received by three beneficiaries in the fields of biology, political science, and medicine, one of which was for three years. Maintaining official affiliations is usually associated with maintaining access to a unit’s infrastructure, especially its IT systems and library. Another seven people mentioned maintaining access to the resources of their host institutions after their visits, so it can be assumed that in these cases, there was also some form of maintaining foreign affiliations. In total, 10 beneficiaries reported maintaining access to the resources of their host units or affiliations to these units. In the case of six people, this provided opportunities to conduct research using unique equipment unavailable in their home institutions or even in Poland. Another three people indicated they would maintain access to electronic resources, disk space, and a computing cluster.

Some reports described formalizations of collaborations between the home and host institutions. Seven reports contained explicit declarations in this respect. In addition to statements regarding signed or planned general collaboration agreements, more detailed declarations sometimes appeared, indicating, for example, new agreements within the Erasmus+ initiative. It should be noted that approximately one in five beneficiaries mentioned joint initiatives in the teaching field (these usually concerned plans to initiate or develop student and doctoral student exchanges). Therefore, some form of collaboration formalization was planned by about a quarter of the beneficiaries. However, since the beneficiary reports mostly mentioned planned formalization or planned joint teaching initiatives, the actual number of effects in the form of organizational collaboration agreements may have been much lower than the relatively high number of declarations suggests.

A tangible manifestation of continued collaboration was the reciprocal visits to Poland by collaborators with whom the beneficiaries became acquainted during their trips abroad. Such an effect appeared in the reports of 22 beneficiaries. These were usually return visit plans, but sometimes there was information about return visits that had already taken place (this concerned three reports), and in one case, there was information about funding for such a return visit that had already been obtained. In this case, the funding was to come from the Ulam NAWA Programme. It is worth underlining that in several other reports, the Ulam Programme was mentioned as a source of financing for possible return visits to Poland. On this basis, it can be concluded that NAWA’s outgoing programs promote its incoming programs.

The beneficiaries also occasionally mentioned other beneficial effects of collaboration, such as acquiring a foreign researcher for the council at a host institution, acquiring a foreign researcher for the board of a journal, or electing a beneficiary to the board of an international association.

#### 5.2.6. Competencies and skills.

Almost all the beneficiaries claimed that their stays abroad strengthened their competencies in the field of scientific research (98.6%), with 90.1% indicating significantly strengthened competencies and 8.5% indicating slightly strengthened competencies. Of the 11 people who mentioned the teaching dimension of their projects, all confirmed that their stays strengthened their competencies in the field of teaching. For two-thirds of the beneficiaries, this impact was significant, and for the other third, it was slight.

Explicit references to the development of competencies developed due to visits to foreign centers appeared in more than 90% of the reports. The beneficiaries mostly emphasized the strengthening of existing competencies or the acquiring of new competences specific to their disciplines, scientific interests, or projects being implemented. This applied to, among other things, laboratory techniques, operating devices, quantitative or qualitative research methods, and programming (specifically, learning R or Python languages appeared quite often). The effects of academic mobility in the form of such specific research competencies were reported by about 73% of the beneficiaries. Most of them described the effect in the form of competencies developed in a given area (e.g., “developing skills in the field of femtosecond spectroscopic techniques”), but some also indicated that their competencies improved as a result of their participation in specialist training (e.g., “during the internship I completed a full eye-tracking course”). Some respondents emphasized that they gained unplanned experience in interdisciplinary research:

It was a chance to conduct slightly different research than I had conducted so far. As an IT specialist, I worked not only on IT tools but also on the analysis of biological data, including those related to SARS-CoV-2 and COVID-19.

The quoted case is especially interesting because it shows not only unintended interdisciplinarity but also that it was an unexpected effect of the pandemic (a response to a crisis situation). Some beneficiaries described their acquisitions of new competencies during their stays abroad as breakthroughs in their professional development:

The implementation of the project was one of the key moments in my academic career. Thanks to the competencies gained, primarily in the field of conducting behavioral experiments, statistics, and programming, I can conduct research that I was not able to conduct before the mobility.

Some beneficiaries expressed their belief that their new competencies and experiences would allow them to improve their future research:

Thanks to the implementation of the project, I managed to introduce a number of new skills and practices used at [a leading university in the USA] to my scientific workshop, and thanks to this, subsequent projects will be at a much higher scientific level, and thus will contribute to the development of the scientific discipline I represent.

About half of the program participants indicated their competencies in planning and implementing research work and other organizational aspects related to research (and occasionally teaching) improved. These competencies included skills in obtaining funding, organizing teamwork (including tools used to manage teams and laboratories), working in teams (including interdisciplinary, multicultural, or spatially dispersed teams) and collaborating with external partners (including international cooperation), leading a research team, acting as a mentor, planning the development of a scientific career, publishing, organizing seminars and other scientific events, teaching related to research, and disseminating research results. The beneficiaries often associated increased competencies in the area of organization and management in science with the prospect of leading their own research groups. A participant who completed a four-month internship at a large public university in the USA provided an example of this:

An important element of the experience was also the opportunity to observe how a very efficiently managed and productive research facility functions, starting from the work culture through to communication, mentoring, database solutions, and extensive internal documentation (instructions, templates, tests, etc.). The observations gathered will be extremely useful in establishing and running my own lab and research team.

Some beneficiaries described specific experiences in the field of organization of research (e.g., regarding publication standards):

During my stay, I also gained extremely valuable experience in the field of publishing in the best journals. First of all, I would mention the standards of conducting work, such as the external registration of hypotheses or systematic reviews in AsPredicted or Prospero and the registration of preprints in databases such as bioRxiv or PsyArXiv.

Approximately every seventh beneficiary claimed that their language skills had improved. This usually concerned the use of English, but two people declared an improvement in their knowledge of Spanish (which was confirmed by exams in both cases), and one person declared an improvement in their knowledge of two languages: German and Russian.

A separate thread worthy of special attention is competence and experience in teaching. Almost every third report referred to these aspects. Only 8.6% of the beneficiaries planned to teach, so for many people, teaching experiences were an additional benefit of their mobility. One of the respondents described this aspect as follows:

Although my stay and activities were of a typically scientific nature, in […] 2019 I also participated in a biology teaching workshops for employees of [the host institution]. The workshops focused on designing classes and methods for verifying acquired knowledge and skills in teaching biology at the academic level.

The beneficiaries listed teaching competency gains, including conducting or co-conducting classes at host institutions (full cycles of classes or short/one-off classes), supervising bachelor’s, master’s, or doctoral theses, academic supervision of students, visiting classes, consultations on teaching, participation in didactic training, familiarization with writing standards, and assessing term papers. Some beneficiaries reported that they used their new competencies in their home institutions by, among others, modifying syllabuses, creating new classes, or even entire new study programs, conducting classes in foreign languages (mainly in English), and involving students in research in novel ways. In the case of some beneficiaries, the effect was ambitious and measurable. This was well illustrated by the statement of a scientist who stayed at a university in the UK:

Observations on the teaching process are extremely valuable to me. A great deal of emphasis is placed on quantitative methods, which are undervalued in political and administrative sciences in Poland. This applies to both research and teaching. […] The experience and knowledge gained are already yielding positive results in my teaching; in the current semester, I am teaching an introduction to the R program, thanks to the courses I attended at [the host university].

Mobility effects in the form of the development of teaching competencies were most often declared by beneficiaries representing the humanities (55.6% of responses) and social sciences (54.1%) and least often by beneficiaries representing natural sciences (only 16.4%). The development of teaching competencies was mentioned by approximately every fourth beneficiary in the case of engineering and technical sciences and every third beneficiary in the case of agricultural, medical, and health sciences.

#### 5.2.7. Careers.

Ten beneficiaries (7%) mentioned career development or promotion among the effects of their projects. These effects encompassed a wide range of situations: receiving job offers from foreign centers (three beneficiaries), obtaining habilitation degrees (two beneficiaries), being promoted to the positions of assistant professor, deputy director of the home institution, head of the laboratory at the home institution, being invited to participate in expert collaboration with a public institution, and becoming a member of the Polish Academy of Sciences.

Another 52 people (37%) indicated that mobility under the Bekker Programme was an important factor in the development of their future careers. In this context, the most frequently mentioned aspect was the contribution of the stay abroad to meeting the criteria for obtaining a habilitation degree or (less often) the position of professor or the academic title of (full) professor.

#### 5.2.8. Frequency of key effects.

**Fig 6:**
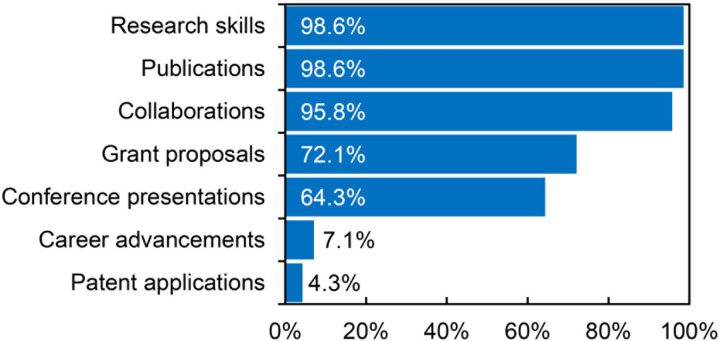
The percentage of beneficiaries declaring specific types of effects.

To summarize the diverse and detailed effects reported by beneficiaries, it is useful to compare the key categories of these outcomes. This overview highlights that nearly all beneficiaries report impacts related to the preparation of publications and the development of scientific competencies. Additionally, 95.8% indicate a strong likelihood of continuing collaboration with the host institution or researchers met during the mobility period. A substantial proportion (72.1%) report preparing new grant applications, while 64.3% cite conference presentations as a direct outcome of their participation. In contrast, career advancement and professional development are reported significantly less frequently, with only 7.1% of beneficiaries indicating such effects, although a notable number anticipate these impacts in the future. The least common outcome is the preparation of patent applications, reported by just 4.3% of beneficiaries (see Fig 6).

#### 5.2.9. Other effects.

Other effects mentioned by one or more beneficiaries were:

- More recognition in the scientific community (several indications),
- Opportunity to collect materials (data, samples) for further research (several indications),
- Collecting literature for further research (several indications),
- The creation of tools for further research (several indications),
- Scientific independence, including independence from supervisors (several indications),
- Enhanced intuition regarding promising research directions,
- Ability to critically evaluate the research and set higher quality standards,
- Increased visibility (“an unexpected effect was a significant increase in my recognition on community portals for scientists (ResearchGate). Before I left for the internship, the total number of reads of my articles during my entire scientific work was less than 8,000; now, it is almost 25,000”),
- An award for the best presentation at a conference,
- The creation or development of a research group,
- A plan to establish a new research center,
- Developing a prototype of a technical solution,
- Designing new research equipment,
- Proposing a doctoral thesis topic and supervision in Poland inspired by the research topics observed at the host institution,
- Enhancing the reputation of the beneficiary as a supervisor of master’s and doctoral theses,
- An outreach lecture,
- A project website,
- A film promoting the project,
- Increase experience and familiarity with R package,
- Promotion of the home institution,
- A better understanding of other cultures, and
- The rebuilding of one’s personal life (“The internship showed me how much I had disrupted the healthy balance between my professional career and private life. It gave me a chance to rebuild many neglected aspects of my personal life”).

Another interesting aspect that emerged from the reports was the opportunity to observe the way science functions in the top institutions of other countries. Beneficiaries described this aspect as “an injection of academic culture,” “participation in the academic life of the host center,” “learning about the culture of work in a leading research center,” “a new perspective on scientific research,” and “the opportunity to participate in an open and well-prepared scientific debate.” One of the beneficiaries provided a more extensive description:

My stay at the host center gave me a different perspective on the work of a scientist. In this center, scientific research is conducted in a different way than I had had the opportunity to carry out before. …[Previously] I had planned my scientific work in detail, predicting the risk of its failure as precisely as possible. Then I carried out the planned tasks. In the center where I stayed, only the research goal was known. Any ideas for its implementation were implemented immediately after their ‘superficial ‘discussion. They were performed with the smallest possible number of repetitions, and then decisions about further actions were made based on the results.

Another scholarship holder emphasized a slightly different aspect:

[I gained insight into] acting in accordance with the “thinking big” philosophy; in other words, developing mainly those fields and branches of research in which the probability of rapid development is the highest, and those that allow for publication in the best journals.

Some respondents stated that they tried to implement in Poland the practices they had learned at the host center: “[The experiences from the host institution] remain an inspiration for me to build a culture of scientific excellence in my current workplace.” Others suggested specific solutions, “I will also try to transfer some of the customs from the host institution to my home institution (e.g., the weekly Journal Club)”.

An interesting case was a beneficiary who reassessed his own research program after observing work in the host institution:

This stay helped me to break through my previous research and analytical patterns, which I had developed in my previous intensive collaborations [...]. As a result, I have abandoned further plans to study [the old topic]. Perhaps I will return to this topic in the future if I manage to find an appropriate anthropological theoretical framework.

## 6. Discussion and conclusions

This article presents the results of an evaluation of the effects of the first call of the Bekker Programme of the Polish National Agency for Academic Exchange, which funds the international mobility of scholars working in Polish research institutions. The study used a mixed research approach incorporating a qualitative analysis of final reports submitted by beneficiaries and a quasi-experimental analysis of publication data, allowing for an estimation of the program’s effects on productivity, scientific impact, and international collaboration. The eight dimensions of academic mobility outcomes defined by Netz et al. served as the analytical framework guiding this study. Moreover, the adoption of a bottom-up coding approach in the qualitative section was deliberately designed to test the utility of this concept—derived from the literature review—in empirical analyses of academic mobility programs’ impacts.The analysis of the final reports submitted by the program beneficiaries indicates a wide range of impacts of foreign mobility funded by the Bekker Programme. Almost all the beneficiaries mentioned effects in the form of preparing publications and developing scientific competencies (including experiencing different organizational cultures) and declared a high probability of continuing collaborations with their host institutions or researchers they met during their stays. Almost three out of four beneficiaries discussed preparing new grant applications. Two-thirds of beneficiaries indicated conference (and other scientific meeting) presentations as an effect of their project implementation, which may exceed the national average for Polish researchers’ international dissemination activity [54] Effects in the form of promotions or scientific career development were much rarer. The preparation of patent applications was a very rarely reported impact. Declarations of effects in the sphere of symbolic capital (recognition in the community, prestige) also appeared sporadically.

The quantitative analysis using interrupted time series indicates no effect on productivity (measured by the number of studies that resulted in published articles) and scientific impact (measured by the average number of citations). This finding aligns with some previous studies, such as those by Bloch [25], which highlight the heterogeneous impacts of research grants, and Gu et al. [17], who observed that mobility might not always translate into immediate productivity gains. However, the regression results regarding the number of internationally co-authored publications are significant, but only when international co-publications are counted fractionally. This outcome suggests that mobility can enhance collaboration networks, as noted by Liu and Hu [18] and Netz et al. [12], but may not uniformly impact other productivity measures.

The result indicating no impact of the grant program is not unheard of in the literature. However, it should be noted that the literature is dominated by studies reporting positive effects (see the literature review in the Introduction section). It should also be underlined that this study is based on a relatively small sample (but the full population of beneficiaries of the first call of the program). The coefficients estimated in the regression models indicate a positive (although very small) impact of the program; however, they are statistically insignificant, which may be due to the small sample size. It cannot be ruled out that increasing the research sample size (by including subsequent calls of the Bekker Programme in the analysis) would result in coefficients exceeding the conventional statistical significance thresholds. Nevertheless, it can be concluded that the results from the analyzed sample of the first call of the Bekker Programme do not provide a basis for claiming that the program increased the number of publications and citations of the beneficiaries’ works. However, they indicate a statistically significant impact of the program on international cooperation measured by co-authored publications with persons affiliated with foreign institutions. It is important to note that the qualitative analysis points to a substantial, multifaceted influence of the program on international collaboration. In this respect, the findings from both quantitative and qualitative approaches are in agreement. This stands in contrast to the program’s impact on productivity and scientific impact, where qualitative analysis reveals an effect that is both felt and reported by beneficiaries, yet this effect does not appear to be corroborated by the quantitative (bibliometric) data.

It can be observed that the effects reported by the beneficiaries of the first call of the Bekker Programme cover all eight categories distinguished by Netz et al. [12]. This is an interesting result primarily because the analysis of the reports was conducted using a bottom-up coding approach (i.e., without pre-defined categories). The fact that the bottom-up coding revealed categories consistent with those proposed by the cited authors suggests the validity of their categorization and confirms the adequacy of the present analysis. Furthermore, it should be noted that this analysis drew attention to the aspect of coming into contact with a different (presumably superior) organizational culture and organization of research work. In the Netz et al. categorization, this aspect does not appear separately but appears as part of the “competencies and personality” category. The element of organizational culture may result from the differences between the organizational culture of the scientific semi-periphery and the ones they encountered during their stays in host institutions, which are located in countries in the scientific center and typically constitute the most prestigious institutions worldwide [55,56]. The differences in organizational culture between the scientific semi-periphery and center can significantly influence scholarly productivity and impact [57,58]. On this basis, it can be concluded that the aspect of observing and potentially translating good organizational practices may be critical in the case of mobility from the scientific periphery and semi-periphery countries to countries in the scientific center [59]. However, this aspect goes beyond the scope of this study and requires further analysis.

The main contribution of this study to the literature on the effects of mobility programs is that it shows a wide spectrum of impacts of the analyzed program from the perspective of the subjective experiences and declarations of its beneficiaries, with a simultaneous lack of evidence for statistically significant effects of the program on productivity measured by the number of publications and scientific impact measured by citations. On the one hand, this may suggest that simple productivity and impact measures do not allow for a full understanding of the complexity of the impacts of mobility. On the other hand, it cannot be ruled out that the declarations may be exaggerated. This study demonstrates the value of combining quantitative and qualitative methodologies in assessing the impact of research grants. This approach provides a more comprehensive understanding of the manifold effects such programs might have. Including qualitative information can be a good counterweight to the over-focus on simplifying metrics that fuel the ultimately destructive publish-or-perish culture in academia.

Building on these insights, it is worth underscoring the broader policy implications that emerge when considering the multi-dimensional and often intangible nature of mobility outcomes. In particular, qualitative methods prove vital for capturing effects that go beyond traditional metrics—such as the acquisition of soft skills and exposure to innovative research cultures—that remain difficult to quantify but hold substantial potential for enhancing scholarly practice. Observing and, where feasible, adopting superior organizational approaches experienced abroad may foster the transfer of valuable work methods and management strategies back to the home institution.

To maximize these benefits, organizational, national, and EU-level mobility policies could place greater emphasis on supporting the identification and dissemination of good practices. Rather than expecting short-term visiting researchers to single-handedly transform their institutions, policy measures might include “soft” mechanisms such as: (1) clear guidance before and during the mobility period on the importance of organizational learning; (2) systematic data collection in post-project evaluations to identify and document good practices in institutional culture; (3) development of curated repositories of effective organizational approaches and practical examples; and (4) facilitation of sustained networking among past beneficiaries to promote ongoing exchanges of knowledge and experiences. By integrating such strategies into mobility schemes, policymakers can bolster the individual-level gains reported by participants while ensuring that institutional and systemic improvements become enduring outcomes of academic mobility.

## 7. Limitations and further research

Three limitations of this study should be acknowledged. First, the sample size is relatively small, which may limit the statistical power of the quantitative analysis. However, this limitation is mitigated by the fact that the study covers the entire population of beneficiaries from the first call of the Bekker Programme. As a result, the analysis includes data on all beneficiaries, eliminating potential biases related to sampling selection. Nonetheless, the small sample size increases the risk that some effects may remain undetected due to insufficient statistical power. This limitation obviously apply only to the qualitative part of the analysis.

Second, the qualitative component of the study lacks a control group, which could raise concerns about attributing observed effects to the program. However, it can be argued, that this limitation is of negligible consequence in this case. The main conclusion of the qualitative analysis—that no significant impact on publication output or citations was observed—indicates a continuation of pre-existing trends rather than a program-induced change. The addition of a control group would therefore likely not alter the primary conclusions regarding the no statistically significant impact (it can be assumed that the trend was also continued in the control group).

Third, the qualitative analysis relies on self-reported data from final reports submitted by beneficiaries for grant accountability purposes. Given that these reports serve as formal documentation of project outcomes, they may be subject to social desirability bias, with beneficiaries potentially presenting their experiences in a more favorable light. However, this limitation is counterbalanced by the unique value of these reports as a rare data source in mobility research. Unlike many previous studies that rely on retrospective surveys or interviews, this study benefits from systematically collected reports prepared at the conclusion of the mobility period. These documents provide detailed and structured insights into the experiences and declared impacts of mobility, offering a perspective that is seldom available in the literature.

This study highlights three key areas for further research. First, expanding the dataset to include multiple calls of the Bekker Programme would increase statistical power and allow for subgroup analyses. Second, employing alternative qualitative methods, such as interviews and independent expert assessments, could help triangulate findings and reduce self-reporting bias. Third, the qualitative analysis revealed the importance of good practices in research organization, including project management, mentoring, data-sharing protocols, and institutional support structures. Future research should examine how these practices spread, what barriers exist, and how mobility programs can actively facilitate their adoption in home institutions.

## Supporting information

S1 File Additional regression results. (DOCX)

S2 File Dataset. (XLSX)
