# Supplementary material for: A mixed-methods evaluation of a Polish international researcher mobility grant scheme
Source: PLoS One. 2025 Jun 25;20(6):e0327196. doi: 10.1371/journal.pone.0327196 (PMC12193014; doi:10.1371/journal.pone.0327196)
Supplement: S1 File — (DOCX) [file pone.0327196.s001.docx]

**Supporting Information for:**

**A mixed-methods evaluation of a Polish international researcher mobility grant scheme**

Adam Ploszaj^1^*

^1^ Science Studies Lab & Centre for European Regional and Local Studies EUROREG, University of Warsaw, Warsaw, Poland

* Corresponding author

E-mail: [a.ploszaj@uw.edu.pl](mailto:a.ploszaj@uw.edu.pl) (AP)

Below, two sets of additional regression tables are presented. The first set (Table 1A) offers a robustness check based on data for a shorter window (2015–2022) instead of 2014–2023. As shown, the core findings remain consistent with the main analysis, indicating that limiting the sample period does not alter the overall conclusions.

Subsequently, results from regressions performed on various data subgroups (Tables 2A–9A) are reported to test whether the main effects might differ substantially across distinct groups (e.g., by gender, age, discipline, or productivity class). These subgroup analyses show no evidence of heterogeneity in the examined dimensions, indicating that the relationships remain consistent across different segments of the sample.

**Table 1A. Regression results – robustness check based on data for a shorter period: 2015-2022**

|  | (1) | (2) | (3) | (4) | (5) | (6) | (7) | (8) |
| --- | --- | --- | --- | --- | --- | --- | --- | --- |
| Outcome variable | Papers published | | Internationally co-authored papers | | Average citations per paper in citation windows | | | |
|  | Whole | Fractional | Whole | Fractional | 2-year | 3-year | 4-year | 5-year |
| Treatment | -0.097 | -0.031 | 0.082 | 0.074** | 0.317 | 0.488 | -0.336 | 1.383 |
|  | (0.242) | (0.089) | (0.170) | (0.035) | (0.545) | (1.069) | (1.831) | (2.629) |
| Pub. year | 0.220*** | 0.033 | 0.177*** | 0.019* | 0.386** | 0.871*** | 1.352** | 1.689** |
|  | (0.079) | (0.031) | (0.052) | (0.011) | (0.159) | (0.325) | (0.537) | (0.717) |
| Soc&Hum | -2.623*** | -0.248** | -1.688*** | -0.164*** | -1.554*** | -2.734*** | -4.149** | -4.823** |
|  | (0.609) | (0.117) | (0.563) | (0.050) | (0.487) | (1.004) | (1.657) | (2.354) |
| ARWU10 | 0.687 | 0.050 | 0.438 | 0.024 | 0.822 | 1.634 | 1.974 | 2.118 |
|  | (0.597) | (0.161) | (0.551) | (0.073) | (0.581) | (1.158) | (1.951) | (2.888) |
| Female | -1.388* | -0.299*** | -1.178* | -0.170*** | -0.380 | -0.861 | -1.404 | -1.952 |
|  | (0.730) | (0.113) | (0.714) | (0.049) | (0.509) | (1.029) | (1.655) | (2.451) |
| Age | -0.050 | 0.022*** | -0.061 | 0.004 | 0.043 | 0.097 | 0.164 | 0.211 |
|  | (0.068) | (0.008) | (0.066) | (0.004) | (0.035) | (0.070) | (0.121) | (0.175) |
| Host country  (dummy) | ✓ | ✓ | ✓ | ✓ | ✓ | ✓ | ✓ | ✓ |
| Observations | 1,216 | 1,216 | 1,216 | 1,216 | 1,216 | 1,216 | 1,064 | 912 |

Robust standard errors in parentheses
*** p<0.01, ** p<0.05, * p<0.1

**Table 2A. Regression results based on data subgroup: female only**

|  | (1) | (2) | (3) | (4) | (5) | (6) | (7) | (8) |
| --- | --- | --- | --- | --- | --- | --- | --- | --- |
| Outcome variable | Papers published | | Internationally co-authored papers | | Average citations per paper in citation windows | | | |
|  | Whole | Fractional | Whole | Fractional | 2-year | 3-year | 4-year | 5-year |
| Treatment | -0.117 | -0.015 | 0.046 | 0.061 | 0.639 | 0.421 | -1.708 | -1.396 |
|  | (0.288) | (0.113) | (0.208) | (0.046) | (0.569) | (1.170) | (1.908) | (2.629) |
| Pub. year | 0.206*** | 0.061** | 0.080* | 0.008 | 0.285** | 0.672*** | 1.125*** | 1.482*** |
|  | (0.067) | (0.029) | (0.044) | (0.009) | (0.120) | (0.233) | (0.363) | (0.487) |
| Soc&Hum | -2.320*** | -0.139 | -1.343*** | -0.141*** | -1.712*** | -2.905** | -4.306** | -5.355** |
|  | (0.417) | (0.132) | (0.405) | (0.051) | (0.585) | (1.154) | (1.835) | (2.488) |
| ARWU10 | 1.516* | 0.142 | 1.329 | 0.106 | 0.733 | 0.838 | 0.630 | -0.186 |
|  | (0.848) | (0.157) | (0.966) | (0.114) | (0.943) | (1.861) | (2.991) | (4.139) |
| Female |  |  |  |  |  |  |  |  |
|  |  |  |  |  |  |  |  |  |
| Age | 0.026 | 0.029** | -0.011 | 0.006 | -0.048 | -0.083 | -0.196 | -0.253 |
|  | (0.039) | (0.014) | (0.037) | (0.004) | (0.042) | (0.084) | (0.134) | (0.187) |
| Host country  (dummy) | ✓ | ✓ | ✓ | ✓ | ✓ | ✓ | ✓ | ✓ |
| Observations | 700 | 700 | 700 | 700 | 700 | 630 | 560 | 490 |

Robust standard errors in parentheses
*** p<0.01, ** p<0.05, * p<0.1

**Table 3A. Regression results based on data subgroup: male only**

|  | (1) | (2) | (3) | (4) | (5) | (6) | (7) | (8) |
| --- | --- | --- | --- | --- | --- | --- | --- | --- |
| Outcome variable | Papers published | | Internationally co-authored papers | | Average citations per paper in citation windows | | | |
|  | Whole | Fractional | Whole | Fractional | 2-year | 3-year | 4-year | 5-year |
| Treatment | -0.032 | -0.065 | 0.170 | 0.063 | 0.639 | 0.421 | -1.708 | -1.396 |
|  | (0.372) | (0.112) | (0.285) | (0.042) | (0.569) | (1.170) | (1.908) | (2.629) |
| Pub. year | 0.229*** | 0.042 | 0.235*** | 0.048*** | 0.285** | 0.672*** | 1.125*** | 1.482*** |
|  | (0.077) | (0.028) | (0.063) | (0.012) | (0.120) | (0.233) | (0.363) | (0.487) |
| Soc&Hum | -2.690* | -0.212 | -2.130 | -0.190** | -1.712*** | -2.905** | -4.306** | -5.355** |
|  | (1.402) | (0.164) | (1.336) | (0.075) | (0.585) | (1.154) | (1.835) | (2.488) |
| ARWU10 | -0.237 | -0.144 | -0.265 | -0.073 | 0.733 | 0.838 | 0.630 | -0.186 |
|  | (0.637) | (0.200) | (0.502) | (0.070) | (0.943) | (1.861) | (2.991) | (4.139) |
| Female |  |  |  |  |  |  |  |  |
|  |  |  |  |  |  |  |  |  |
| Age | -0.083 | 0.016* | -0.075 | 0.004 | -0.048 | -0.083 | -0.196 | -0.253 |
|  | (0.084) | (0.009) | (0.082) | (0.004) | (0.042) | (0.084) | (0.134) | (0.187) |
| Host country  (dummy) | ✓ | ✓ | ✓ | ✓ | ✓ | ✓ | ✓ | ✓ |
| Observations | 820 | 820 | 820 | 820 | 820 | 738 | 656 | 574 |

Robust standard errors in parentheses
*** p<0.01, ** p<0.05, * p<0.1

**Table 4A. Regression results based on data subgroup: younger scholars (up to 37 years old)**

|  | (1) | (2) | (3) | (4) | (5) | (6) | (7) | (8) |
| --- | --- | --- | --- | --- | --- | --- | --- | --- |
| Outcome variable | Papers published | | Internationally co-authored papers | | Average citations per paper in citation windows | | | |
|  | Whole | Fractional | Whole | Fractional | 2-year | 3-year | 4-year | 5-year |
| Treatment | 0.096 | 0.113 | 0.092 | 0.073* | -0.172 | -0.645 | -1.910 | -0.955 |
|  | (0.330) | (0.095) | (0.295) | (0.042) | (0.806) | (1.598) | (2.749) | (3.817) |
| Pub. year | 0.210*** | 0.024 | 0.209*** | 0.036*** | 0.377** | 0.731** | 1.160** | 1.393** |
|  | (0.077) | (0.026) | (0.062) | (0.011) | (0.187) | (0.343) | (0.542) | (0.701) |
| Soc&Hum | -0.586 | -0.055 | 0.049 | -0.122 | -2.352* | -3.779* | -5.096* | -5.831 |
|  | (1.965) | (0.173) | (1.855) | (0.089) | (1.278) | (2.273) | (3.022) | (4.184) |
| ARWU10 | 0.129 | 0.089 | 0.021 | 0.050 | 1.908* | 3.422 | 4.165 | 4.694 |
|  | (1.261) | (0.160) | (1.439) | (0.114) | (1.157) | (2.220) | (3.408) | (5.060) |
| Female | -0.370 | -0.213 | -0.212 | -0.134*** | -0.417 | -1.020 | -1.102 | -0.426 |
|  | (0.777) | (0.151) | (0.819) | (0.051) | (0.863) | (1.726) | (2.723) | (4.020) |
| Age | -1.051 | -0.031 | -0.962 | -0.001 | 0.362 | 0.674 | 0.687 | 1.247 |
|  | (0.855) | (0.038) | (0.874) | (0.016) | (0.321) | (0.568) | (0.703) | (0.965) |
| Host country  (dummy) | ✓ | ✓ | ✓ | ✓ | ✓ | ✓ | ✓ | ✓ |
| Observations | 770 | 770 | 770 | 770 | 770 | 693 | 616 | 539 |

Robust standard errors in parentheses
*** p<0.01, ** p<0.05, * p<0.1

**Table 5A. Regression results based on data subgroup: younger scholars (over 37 years old)**

|  | (1) | (2) | (3) | (4) | (5) | (6) | (7) | (8) |
| --- | --- | --- | --- | --- | --- | --- | --- | --- |
| Outcome variable | Papers published | | Internationally co-authored papers | | Average citations per paper in citation windows | | | |
|  | Whole | Fractional | Whole | Fractional | 2-year | 3-year | 4-year | 5-year |
| Treatment | -0.243 | -0.201 | 0.133 | 0.052 | 1.299*** | 2.630*** | 2.727 | 5.875 |
|  | (0.350) | (0.126) | (0.207) | (0.046) | (0.403) | (1.004) | (2.139) | (4.103) |
| Pub. year | 0.227*** | 0.078** | 0.117** | 0.023** | 0.260*** | 0.525*** | 0.826*** | 0.948** |
|  | (0.068) | (0.031) | (0.049) | (0.011) | (0.093) | (0.188) | (0.291) | (0.403) |
| Soc&Hum | -2.229*** | -0.285** | -1.279*** | -0.214*** | -1.382** | -2.195* | -3.266 | -2.891 |
|  | (0.394) | (0.141) | (0.294) | (0.055) | (0.570) | (1.207) | (2.049) | (2.732) |
| ARWU10 | 0.142 | -0.101 | 0.019 | -0.054 | 0.169 | 0.213 | -0.226 | -0.726 |
|  | (0.640) | (0.219) | (0.449) | (0.091) | (0.706) | (1.471) | (2.434) | (3.275) |
| Female | -0.629 | -0.311** | -0.323 | -0.114* | 0.076 | 0.520 | 0.662 | 0.516 |
|  | (0.421) | (0.141) | (0.292) | (0.068) | (0.497) | (1.059) | (1.804) | (2.319) |
| Age | -0.005 | 0.015 | -0.010 | -0.003 | 0.043 | 0.108 | 0.208 | 0.291 |
|  | (0.030) | (0.010) | (0.023) | (0.005) | (0.052) | (0.097) | (0.170) | (0.231) |
| Host country  (dummy) | ✓ | ✓ | ✓ | ✓ | ✓ | ✓ | ✓ | ✓ |
| Observations | 750 | 750 | 750 | 750 | 750 | 675 | 600 | 525 |

Robust standard errors in parentheses
*** p<0.01, ** p<0.05, * p<0.1

**Table 6A. Regression results based on data subgroup: scholars in social sciences and humanities**

|  | (1) | (2) | (3) | (4) | (5) | (6) | (7) | (8) |
| --- | --- | --- | --- | --- | --- | --- | --- | --- |
| Outcome variable | Papers published | | Internationally co-authored papers | | Average citations per paper in citation windows | | | |
|  | Whole | Fractional | Whole | Fractional | 2-year | 3-year | 4-year | 5-year |
| Treatment | -0.438 | -0.275* | -0.112 | -0.008 | 1.173** | 1.210 | 0.291 | -0.336 |
|  | (0.278) | (0.143) | (0.125) | (0.044) | (0.536) | (1.352) | (2.371) | (2.938) |
| Pub. year | 0.212*** | 0.089** | 0.112** | 0.024* | 0.154 | 0.382* | 0.679 | 1.024* |
|  | (0.064) | (0.036) | (0.048) | (0.013) | (0.098) | (0.221) | (0.415) | (0.573) |
| Soc&Hum |  |  |  |  |  |  |  |  |
|  |  |  |  |  |  |  |  |  |
| ARWU10 | 0.049 | -0.024 | 0.124 | 0.018 | 0.669 | 1.203 | 1.355 | 0.044 |
|  | (0.541) | (0.239) | (0.346) | (0.084) | (0.707) | (1.612) | (2.795) | (3.998) |
| Female | -0.604 | -0.112 | -0.267 | -0.068 | -0.505 | -1.022 | -1.514 | -1.558 |
|  | (0.484) | (0.151) | (0.309) | (0.073) | (0.633) | (1.377) | (2.382) | (3.169) |
| Age | -0.040 | -0.006 | -0.026 | -0.002 | 0.085 | 0.183 | 0.347 | 0.380 |
|  | (0.054) | (0.014) | (0.030) | (0.007) | (0.069) | (0.160) | (0.290) | (0.408) |
| Host country  (dummy) | ✓ | ✓ | ✓ | ✓ | ✓ | ✓ | ✓ | ✓ |
| Observations | 520 | 520 | 520 | 520 | 520 | 468 | 416 | 364 |

Robust standard errors in parentheses
*** p<0.01, ** p<0.05, * p<0.1

**Table 7A. Regression results based on data subgroup: scholars not from social sciences and humanities**

|  | (1) | (2) | (3) | (4) | (5) | (6) | (7) | (8) |
| --- | --- | --- | --- | --- | --- | --- | --- | --- |
| Outcome variable | Papers published | | Internationally co-authored papers | | Average citations per paper in citation windows | | | |
|  | Whole | Fractional | Whole | Fractional | 2-year | 3-year | 4-year | 5-year |
| Treatment | 0.120 | 0.080 | 0.229 | 0.099** | 0.232 | 0.847 | 0.424 | 3.845 |
|  | (0.334) | (0.093) | (0.266) | (0.041) | (0.635) | (1.275) | (2.368) | (3.982) |
| Pub. year | 0.222*** | 0.031 | 0.191*** | 0.033*** | 0.405*** | 0.758*** | 1.160*** | 1.251** |
|  | (0.071) | (0.024) | (0.055) | (0.010) | (0.151) | (0.275) | (0.418) | (0.542) |
| Soc&Hum |  |  |  |  |  |  |  |  |
|  |  |  |  |  |  |  |  |  |
| ARWU10 | 0.850 | 0.008 | 0.558 | -0.003 | 1.137 | 1.920 | 2.429 | 3.351 |
|  | (0.926) | (0.169) | (0.936) | (0.104) | (0.805) | (1.506) | (2.401) | (3.674) |
| Female | -1.680 | -0.343** | -1.423 | -0.160*** | -0.378 | -0.823 | -1.107 | -2.916 |
|  | (1.142) | (0.142) | (1.156) | (0.056) | (0.693) | (1.399) | (2.255) | (3.487) |
| Age | -0.056 | 0.030*** | -0.076 | 0.007** | 0.057 | 0.101 | 0.168 | 0.242 |
|  | (0.094) | (0.009) | (0.095) | (0.003) | (0.042) | (0.078) | (0.128) | (0.196) |
| Host country  (dummy) | ✓ | ✓ | ✓ | ✓ | ✓ | ✓ | ✓ | ✓ |
| Observations | 1,000 | 1,000 | 1,000 | 1,000 | 1,000 | 900 | 800 | 700 |

Robust standard errors in parentheses
*** p<0.01, ** p<0.05, * p<0.1

**Table 8A. Regression results based on data subgroup: more productive scholar (above the median number of publications in the period 2014-2018)**

|  | (1) | (2) | (3) | (4) | (5) | (6) | (7) | (8) |
| --- | --- | --- | --- | --- | --- | --- | --- | --- |
| Outcome variable | Papers published | | Internationally co-authored papers | | Average citations per paper in citation windows | | | |
|  | Whole | Fractional | Whole | Fractional | 2-year | 3-year | 4-year | 5-year |
| Treatment | -0.210 | -0.020 | 0.137 | 0.096* | 0.909 | 1.754 | 0.927 | 3.044 |
|  | (0.421) | (0.126) | (0.332) | (0.051) | (0.620) | (1.230) | (2.194) | (3.202) |
| Pub. year | 0.276*** | 0.039 | 0.246*** | 0.039*** | 0.281** | 0.525** | 0.861** | 1.007* |
|  | (0.089) | (0.030) | (0.073) | (0.013) | (0.132) | (0.246) | (0.376) | (0.524) |
| Soc&Hum | -2.631* | 0.243 | -2.235 | -0.022 | -1.589 | -2.932 | -4.422 | -6.497 |
|  | (1.525) | (0.208) | (1.607) | (0.089) | (1.120) | (2.222) | (3.582) | (4.401) |
| ARWU10 | 1.750 | 0.035 | 1.425 | 0.044 | 0.915 | 1.232 | 1.051 | 0.967 |
|  | (1.075) | (0.179) | (1.290) | (0.118) | (0.930) | (1.820) | (3.008) | (4.183) |
| Female | -2.547 | -0.406** | -2.133 | -0.220*** | 0.037 | 0.598 | 1.765 | 2.541 |
|  | (1.549) | (0.178) | (1.593) | (0.071) | (0.776) | (1.497) | (2.227) | (3.294) |
| Age | -0.150 | 0.012 | -0.136 | 0.000 | -0.014 | -0.057 | -0.097 | -0.180 |
|  | (0.104) | (0.008) | (0.108) | (0.004) | (0.046) | (0.079) | (0.126) | (0.150) |
| Host country  (dummy) | ✓ | ✓ | ✓ | ✓ | ✓ | ✓ | ✓ | ✓ |
| Observations | 790 | 790 | 790 | 790 | 790 | 711 | 632 | 553 |

Robust standard errors in parentheses
*** p<0.01, ** p<0.05, * p<0.1

**Table 9A. Regression results based on data subgroup: less productive scholar (below the median number of publications in the period 2014-2018)**

|  | (1) | (2) | (3) | (4) | (5) | (6) | (7) | (8) |
| --- | --- | --- | --- | --- | --- | --- | --- | --- |
| Outcome variable | Papers published | | Internationally co-authored papers | | Average citations per paper in citation windows | | | |
|  | Whole | Fractional | Whole | Fractional | 2-year | 3-year | 4-year | 5-year |
| Treatment | 0.079 | -0.065 | 0.086 | 0.026 | 0.170 | 0.124 | -0.215 | 1.734 |
|  | (0.207) | (0.095) | (0.113) | (0.034) | (0.675) | (1.480) | (2.784) | (4.722) |
| Pub. year | 0.156*** | 0.064** | 0.075*** | 0.019*** | 0.360** | 0.742** | 1.141** | 1.353** |
|  | (0.047) | (0.026) | (0.023) | (0.007) | (0.167) | (0.311) | (0.501) | (0.630) |
| Soc&Hum | -0.651*** | 0.077 | -0.334** | -0.037 | -2.113*** | -3.825*** | -5.836*** | -7.423** |
|  | (0.146) | (0.063) | (0.135) | (0.035) | (0.549) | (1.173) | (2.026) | (3.153) |
| ARWU10 | -0.125 | -0.075 | -0.033 | -0.025 | 0.874 | 1.830 | 2.417 | 2.980 |
|  | (0.250) | (0.139) | (0.179) | (0.050) | (0.778) | (1.686) | (2.909) | (4.393) |
| Female | -0.224 | -0.122* | -0.165 | -0.056** | -1.280** | -3.071** | -5.247** | -7.852** |
|  | (0.142) | (0.066) | (0.115) | (0.028) | (0.610) | (1.296) | (2.239) | (3.438) |
| Age | 0.007 | 0.007 | 0.007 | 0.002 | 0.174** | 0.409** | 0.701** | 1.212** |
|  | (0.018) | (0.009) | (0.012) | (0.003) | (0.071) | (0.180) | (0.356) | (0.585) |
| Host country  (dummy) | ✓ | ✓ | ✓ | ✓ | ✓ | ✓ | ✓ | ✓ |
| Observations | 730 | 730 | 730 | 730 | 730 | 657 | 584 | 511 |

Robust standard errors in parentheses
*** p<0.01, ** p<0.05, * p<0.1
